# Supplementary figures and images for: Necroptosis-Related Gene Signature Predicts Prognosis in Patients with Advanced Ovarian Cancer
Source: Cancers (Basel). 2025 Jan 15;17(2):271. doi: 10.3390/cancers17020271 (PMC11763378; doi:10.3390/cancers17020271)

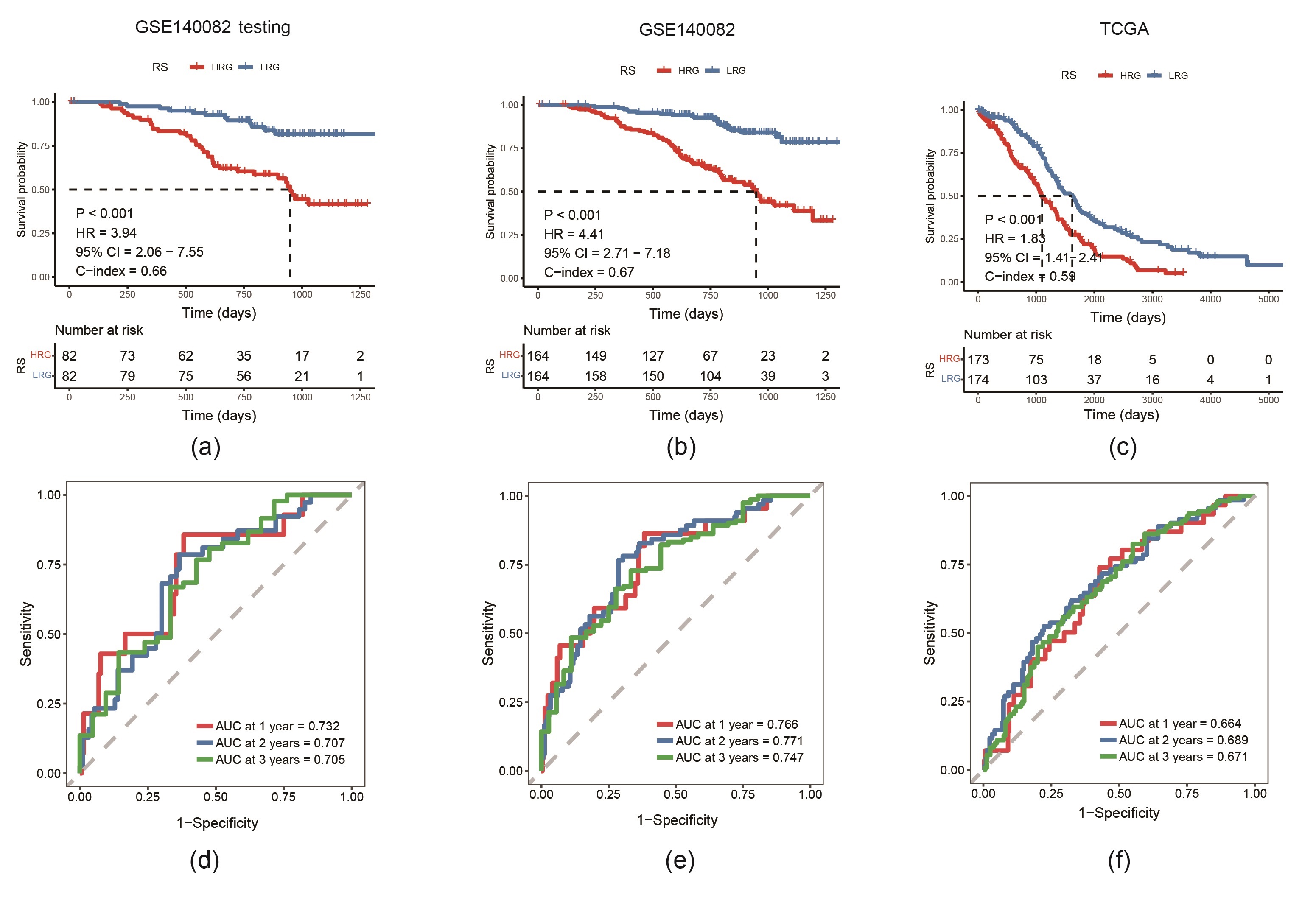

Supplement: Supplementary file 1 [file cancers-17-00271-s001.zip › Figure_S1.jpg]

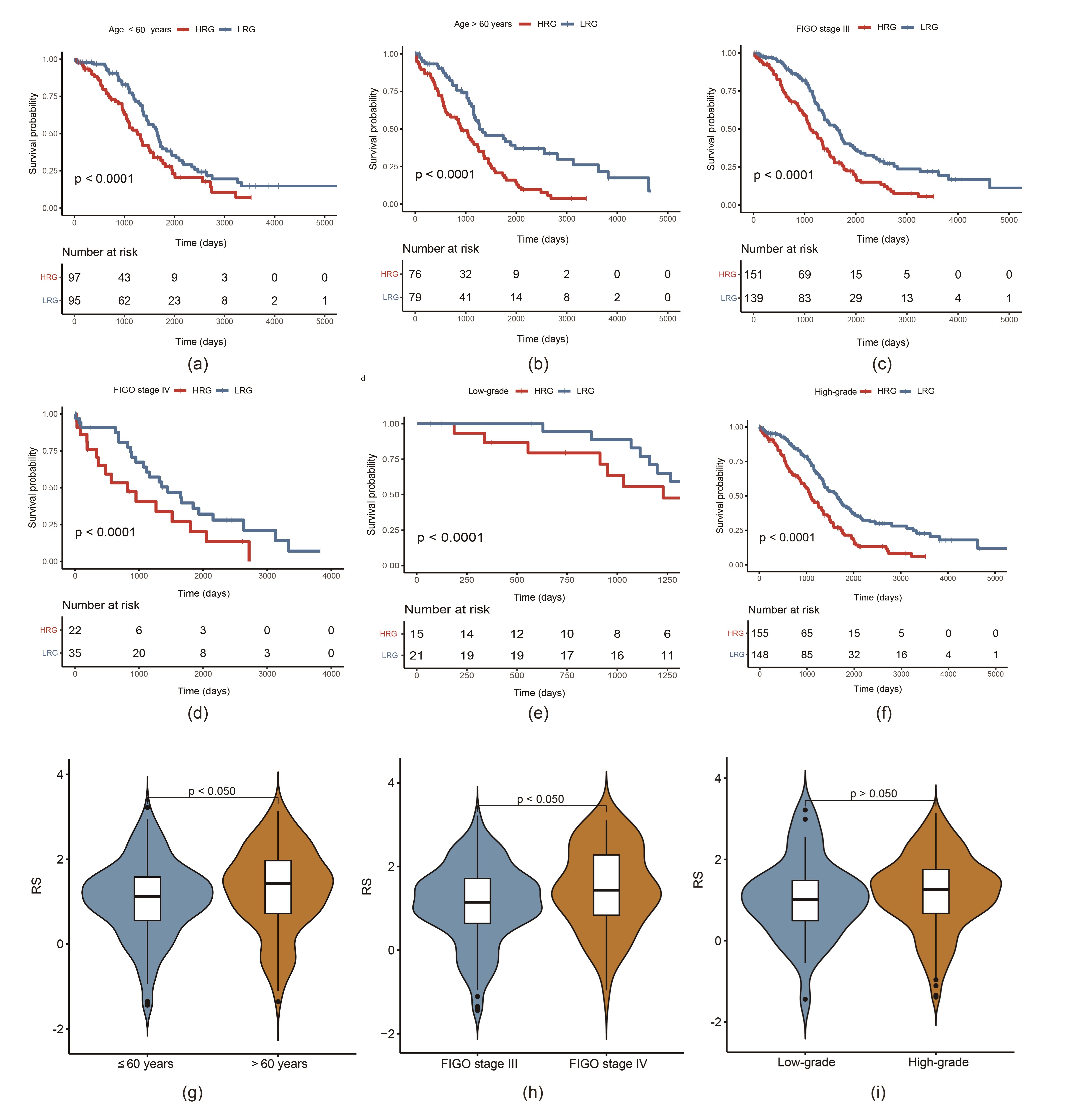

Supplement: Supplementary file 1 [file cancers-17-00271-s001.zip › Figure_S2.jpg]

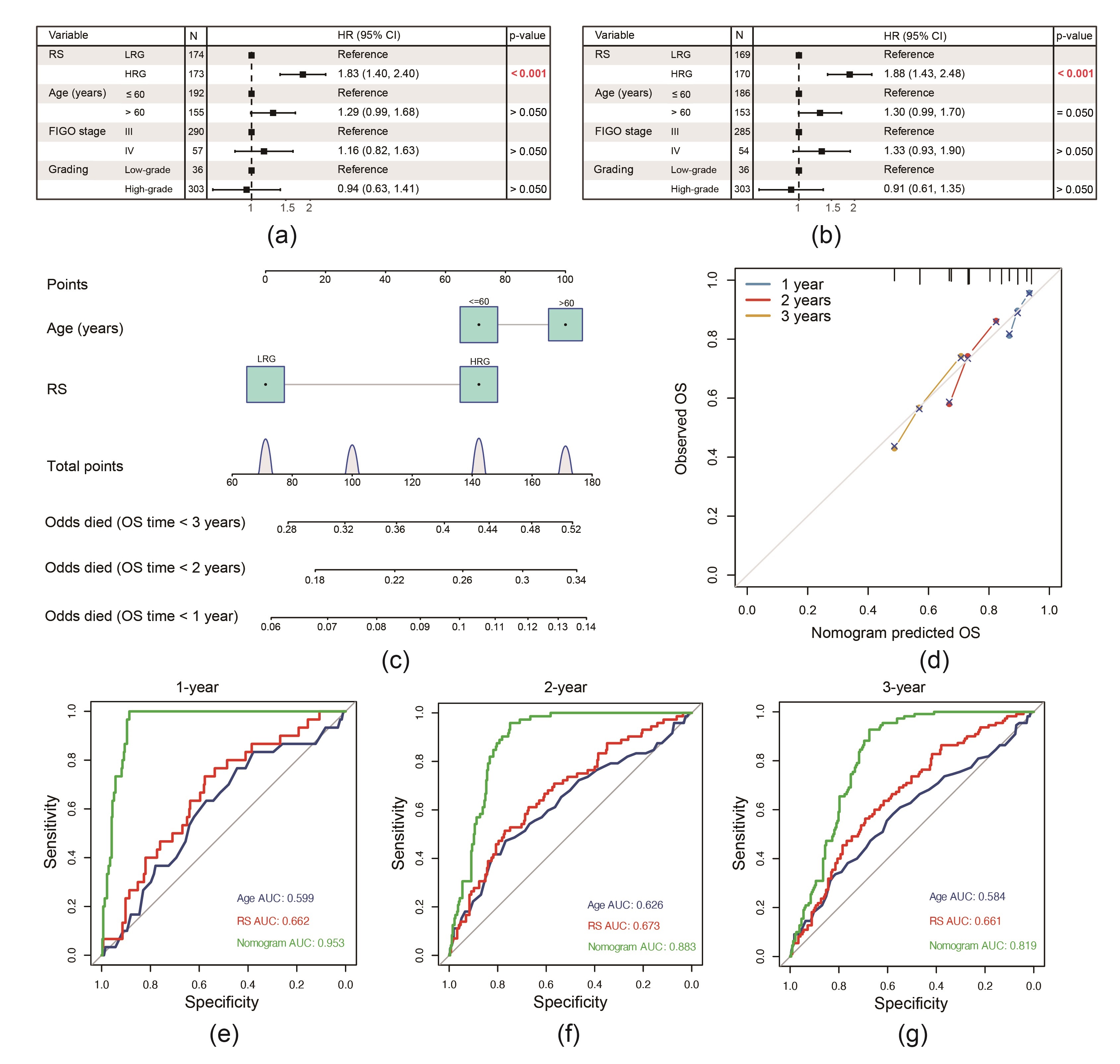

Supplement: Supplementary file 1 [file cancers-17-00271-s001.zip › Figure_S3.jpg]
